# Effect of Zhuyun recipe on endometrial pinopode expression in mice with embryonic implantation dysfunction and ovulation stimulation

**DOI:** 10.3892/etm.2014.2138

**Published:** 2014-12-16

**Authors:** NAN YU, WENJIE YAN, YAQIN WANG, TAILANG YIN, YUE GUO, JING YANG

**Affiliations:** 1 Department of Obstetrics and Gynecology, Tongji Hospital, Tongji Medical College, Huazhong University of Science and Technology, Wuhan, Hubei 430030, P.R. China; 2 Reproductive Medical Center, Renmin Hospital of Wuhan University, Wuhan, Hubei 430060, P.R. China

**Keywords:** ovarian stimulation, embryo implantation dysfunction, traditional Chinese medicine, endometrial receptivity, pinopodes, mouse

## Abstract

Zhuyun recipe (ZYR) is a traditional Chinese medicine that has been widely used as an infertility treatment for a number of years. Although the therapeutic effects are desirable and satisfactory, the therapeutic mechanism of ZYR remains poorly understood. In the present study, pinopodes were investigated as an important morphological marker of endometrial receptivity, in order to further investigate the therapeutic mechanism of ZYR. The expression of pinopodes during the implantation window was observed using scanning electron microscopy in mice with induced ovarian stimulation (OS) and embryo implantation dysfunction (EID). A marked decrease in the number of fully developed pinopodes was observed on the endometrial surface in the OS and EID model mice, which was in accordance with the decreased pregnancy rate and number of embryonic implantation sites when compared with the control. Following treatment with ZYR, the spatial and temporal expression of pinopodes in the OS and EID mice was found to be similar to the control mice. In conclusion, ZYR was demonstrated to improve endometrial receptivity in OS and EID mice through significant improvements in the spatial and temporal expression of pinopodes.

## Introduction

Embryo implantation is a key factor for a successful pregnancy. In a previous study, Boomsma *et al* (1) demonstrated that the contribution of implantation failure in the number of unsuccessful pregnancies was higher in stimulated cycles (50%) compared with natural cycles (30%). Therefore, the embryo quality and the status of the endometrium are considered to be very important for achieving good clinical outcomes in patients undergoing assisted reproductive technology (ART) treatment (2). ART involves a dynamic and complex process of interaction and inter-acceptance between the embryo and matrix. A key step is the synchronization of the receptive endometrium with the blastocyst at a certain developmental stage, which has the ability to accept the endometrium (3). According to a number of animal experiments, the time period during which the endometrium can accept a blastocyst implantation, known as the implantation window, is limited and lasts only for a few hours in rodents (4,5). Similarly, the human implantation window appears to be between days 20 and 21 of the menstrual cycle, which is consistent with the appearance of pinopodes on the surface of endometrial epithelial cells (6,7). Furthermore, a positive correlation between the number of pinopodes and embryo implantation has been reported. Therefore, pinopodes are recognized as an ultrastructural marker of endometrial receptivity (3).

Zhuyun recipe (ZYR) is a traditional Chinese medicine that has been widely used for infertility treatment and *in vitro* fertilization (IVF) with embryo transfer in clinical practice. Although the application results are desirable and satisfactory, the therapeutic mechanism of ZYR remains unclear (8). In a previous study, ZYR was shown to significantly increase the pregnancy rate and number of embryonic implantation sites, as well as the expression of endometrial leukemia inhibitory factor (LIF) and integrin β3 subunit, which are markers of endometrial receptivity (9). In the present study, the effect of ZYR treatment on pinopode expression was investigated in mice subjected to ovulation stimulation (OS) and embryo implantation dysfunction (EID). The aim of the study was to further explore the effects of ZYR on endometrial receptivity.

## Materials and methods

### Plant material and extract preparation

ZYR consists of *Epimedium brevicornum* Maxim, *Morinda officinalis* How, Chinese Dodder and *Eucommia ulmoides*, and was purchased from the Traditional Chinese Medicine Department of Renmin Hospital of Wuhan University (Wuhan, China). The ZYR formula was developed by our research group led by Professor Yang, and the four aforementioned herbal materials were mixed in a ratio of 15:12:20:15, respectively. An aqueous extract of ZYR was produced using a previously described method (9). Briefly, the four medicinal materials were mixed, macerated for 1 h in 8 volume/weight (v/w) distilled water and decocted for 1 h. The filtrate was collected and the residue was decocted for a further 1 h with 6 v/w distilled water. Next, the filtrates were pooled and concentrated, and the extract was sealed and stored at −20°C. The aseptic decoction contained 0.6 g/ml crude drug.

### Animals

A total of 209 mature Kunming mice of specific-pathogen-free grade (age, 6–8 weeks; weight, 25–28 g) were provided by Wuhan University Laboratory Animal Center (Wuhan, China). The female mice were virginal and the male mice had been proven to be fertile. The mice were bred separately, with free access to water and a standard diet. The animals were housed in the laboratory on a 12 h light/dark regimen, at 18–22°C and 70–85% relative humidity. The study protocol was conformed to the Guide for the Care and Use of Laboratory Animals published by the National Institutes of Health (NIH publication no. 85-23, revised 1996), and was approved by the Ethics Committee of Wuhan University. The animals were handled according to the Wuhan Directive for Animal Research.

### Animal models and experimental protocol

All 139 female mice were randomly divided into six groups, including the control (22 mice), OS model (19 mice), OS + ZYR (26 mice), EID model (20 mice), EID + ZYR (27 mice) and ZYR only (25 mice) groups. OS was induced in the OS and OS + ZYR groups with an intraperitoneal injection of 40 IU/100 g pregnant mare serum gonadotropin (PMSG, Hangzhou Yunuo Chemical Co., Ltd, Hangzhou, China), followed by 100 IU/100 g human chorionic gonadotropin (HCG, Lizhu Pharmaceutical Trading Co., Ltd, Zhuhai, China) after 48 h. After 3 h, the female mice were caged with the male mice (ratio, 2:1) overnight and the mice were checked for the presence of a vaginal plug the following morning. Presence of a vaginal plug was considered as evidence of successful mating, designating the day as day 1 postcoitum. To induce the EID model, mice in the EID and EID + ZYR groups were injected subcutaneously with 0.1 ml mifepristone dissolved in 0.08 mg/0.1 ml propanediol (mifepristone, Hubei Gedian Renfu Pharmaceutical Co., Ltd, Ezhou, China) on day 4 postcoitum (10).

In the three ZYR groups (OS + ZYR, EID + ZYR and ZYR only), the female mice received a daily gastric perfusion of ZYR decoction during the first four days of pregnancy, with an oral administration volume of 1.5 ml/100 g per day. The OS and EID model groups received the same amount of saline, while the control group were not treated.

Two mice from each group were sacrificed by cervical dislocation at 21:30–22:00 of day 4 postcoitum, regarded as time point 1 (T1), while two further mice from each group were sacrificed at 09:30–10:00 of day 5 postcoitum (T2). The uterine horns were removed for scanning electron microscopic observation (Type S_520; Hitachi Instruments, Inc., Tokyo, Japan). The remaining female mice were sacrificed on day 8 postcoitum and the uterine horns were excised to determine the number of implantation sites. The number of pregnant mice and corresponding implanted embryos in each uterine was recorded.

### Ultrastructural observation

Murine uterine horns were cut open along the longitudinal axis, rinsed with saline solution and fixed in sodium cacodylate buffer (0.15 mol/l, pH 7.3), containing 2.5% w/v glutaraldehyde. Next, the samples were refixed in 1% w/v osmium tetroxide, dehydrated in a graded series of acetone, dried in a critical-point dryer, mounted on a specimen holder and coated with gold palladium. A scanning electron microscope (Type S-520; Hitachi Instruments) was used to observe the samples.

All observations were performed by an appointed observer. The pinopodes were divided according to their developmental stage into developing, fully developed and regressing pinopodes. In addition, the samples were divided into three grades, based on the number of pinopodes present (11,12): Few, when <20% pinopodes were observed in the whole area of the endometrium; moderate, when 20–50% pinopodes were detected; and abundant, when >50% pinopodes were observed.

### Statistical analysis

Data were statistically analyzed and are expressed as the mean ± standard error of mean. The mean values were analyzed by analysis of variance, followed by the Newman-Keuls test, while the number of pregnant mice was analyzed by the χ^2^ test. Data analysis was performed using SPSS version 17.0 (SPSS Inc., Chicago, IL, USA). P<0.05 was considered to indicate a statistically significant difference.

## Results

### Comparison of the pregnancy rate and number of implantation sites in the pregnant mice

**Figure 1:**
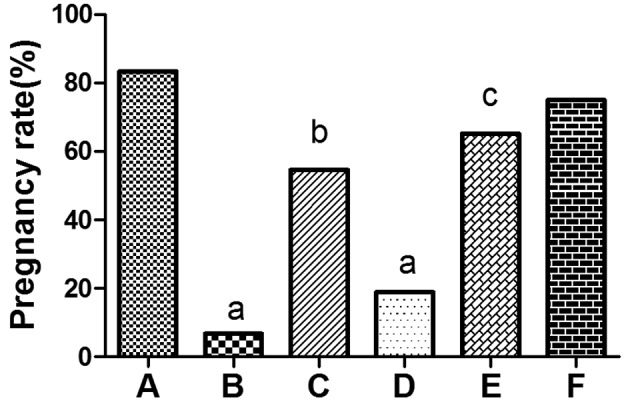
Pregnancy rate on day 8 postcoitum in (A) control, (B) OS model, (C) OS + ZYR, (D) EID model, (E) EID + ZYR and (F) ZYR only groups. ^a^P<0.001, vs. control group; ^b^P<0.01, vs. OS group; ^c^P<0.01, vs. EID group. OS, ovulation stimulation; ZYR, Zhuyun recipe; EID, embryo implantation dysfunction.

The pregnancy rates in the OS (6.67%) and EID (18.75%) model groups were significantly lower compared with the control group (83.33%; P<0.001). However, the pregnancy rates of the OS + ZYR (54.55%) and EID + ZYR (65.22%) groups were evidently increased compared with the corresponding model groups (OS and EID, respectively; P<0.01; Fig. 1). The number of implanted embryos in the EID group (6.67±1.16) was lower compared with the control group (13.80±1.42; P<0.01). However, following ZYR treatment, the number of implanted embryos in the EID + ZYR group (10.13±3.18) was higher compared with the EID group (P<0.05). By contrast, no statistically significant differences were observed in the pregnancy rate and number of implanted embryos between the ZYR only and control groups.

### Pinopode expression on the endometrial surface during the implantation window

Murine embryo implantation normally initiates between 10:00 and 22:00 on day 4 postcoitum, while the implantation window is maintained between the afternoon of day 4 postcoitum and the morning of day 5 postcoitum. In order to understand the establishment of endometrial receptivity during the window of implantation, two time-points were selected (T1, 21:30–22:00 of day 4 postcoitum; T2, 09:30–10:00 of day 5 postcoitum) for the observation of pinopode expression.

**Figure 2:**
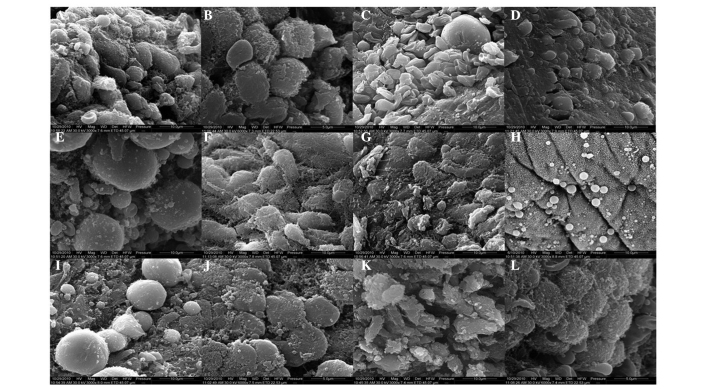
Scanning electron microscopy images showing the expression of pinopodes on the endometrial surface during the implantation window of mice. In the control group, (A) developing pinopodes at T1 and (B) fully developed pinopodes at T2 were observed. In the OS group, (C) regressing pinopodes at T1 and (D) fully regressing pinopodes at T2 were observed. In the OS + ZYR group, (E) a number of fully developed pinopodes at T1 and (F) developing pinopodes at T2 were observed. In the EID group, (G) restrained expression of pinopodes at T1 and (H) fully restrained expression of pinopodes at T2 were observed. In the EID + ZYR group, (I) a number of fully developed pinopodes at T1 and (J) fully developed pinopodes at T2 were observed. In the ZYR only group, (K) irregularly developing pinopodes at T1 and (L) fully developed pinopodes at T2 were observed. Magnification for a, c-i and k, ×3,000; magnification for b, j and l, ×6,000. OS, ovulation stimulation; EID, embryo implantation dysfunction; ZYR, Zhuyun recipe.

In the control group, specimens collected at T1 revealed a large number of membranous projections, with inconsistent shapes and sizes, unclear boundaries and rough surfaces covered with short microvilli, which indicated the presence of abundant developing pinopodes (Fig. 2A). At T2, the abundant pinopodes were evenly distributed over the endometrial surface, more protruded and consistent in shape and size, and had clearer boundaries, while the microvilli merged together and disappeared gradually, indicating abundant fully developed pinopodes (Fig. 2B).

In the OS group, a moderate number of pinopodes without microvilli were observed on the endometrial surface at T1, which were mostly collapsed and flat, representing moderate regressing pinopodes (Fig. 2C). At T2, few, small and scattered pinopodes were distributed over the endometrial surface (Fig. 2D), which appeared earlier compared with the control group. By contrast, in the OS + ZYR group, moderate pinopodes of different sizes were observed on the endometrial surface at T1, a number of which were well-developed with large projections (Fig. 2E). At T2, a greater number of cell surfaces showed projections, which represented abundant developing pinopodes (Fig. 2F). When compared with the OS group, the appearance of pinopodes on the endometrial surface was delayed following treatment with ZYR, which was comparable to the control group.

In the EID group, few pinopodes with microvilli appeared on the endometrial surface at T1 (Fig. 2G). However, at T2, fewer well-developed pinopodes with small projections were observed (Fig. 2H), indicating that pinopode appearance was restrained by mifepristone. In the EID + ZYR group, moderate pinopodes of different sizes were unevenly distributed over the endometrial surface at T1, a number of which were fully developed with large projections (Fig. 2I). At T2, abundant fully developed pinopodes were observed (Fig. 2J). Therefore, the restrained expression of pinopodes in the EID group was improved following treatment with ZYR, and the expression was similar to the control group.

In the ZYR only group, the majority of the microvilli had disappeared at T1 and the pinopodes were transformed into irregularly shaped projections, without distinguished cell borders (Fig. 2K). At T2, the pinopodes appeared to be well-developed, although a few had collapsed (Fig. 2L), and were similar to the pinopodes observed in the control group.

## Discussion

In the present study, the effect of ZYR on the expression of pinopodes on the endometrial surface of mice subjected to ovarian stimulation (OS) or embryo implantation dysfunction (EID) was demonstrated for the first time. A marked decrease in the number of fully developed pinopodes on the endometrial surface was observed in the OS and EID model mice, which was in accordance with the decreased pregnancy rates and embryonic implantation sites of the two model groups, when compared with the control group. Following treatment with ZYR, an evident increase was observed in the expression of fully developed pinopodes in the EID + ZYR and OS + ZYR groups, while the pregnancy rates and embryonic implantation sites of these groups also increased. In a previous study (9), ZYR was found to significantly increase the pregnancy rate and number of embryonic implantation sites, and partly improve endometrial receptivity through reinforcing the expression of endometrial LIF and integrin β3 subunit. In the present study, pinopode expression was investigated to further explore the therapeutic mechanism of ZYR. The pinopodes were found to be fully developed in the OS + ZYR and EID + ZYR groups. Thus, the results of the present study reinforce the hypothesis that the endometrial expression of pinopodes is positively associated with endometrial receptivity and embryonic implantation. In addition, ZYR was found to improve the endometrial receptivity in OS and EID mice by significantly increasing the spatial and temporal expression of pinopodes.

Pinopodes are widely known to be an important morphological marker indicating the establishment of endometrial receptivity and the opening of the implantation window. The appearance and full development of pinopodes indicates that the endometrium is ready for blastocyst adhesion and implantation (7,13,14). Pinopode deficiency in reproductive females undergoing *in vitro* fertilization (IVF) and embryo transfer treatment results in multiple implantation failures. Previous studies in humans have revealed that the window of implantation, which begins upon the formation of fully developed pinopodes, opens and closes earlier in females undergoing ovarian hyperstimulation for IVF compared with females with natural cycles (15–17). In addition, a low dose of mifepristone, which is administered as a progesterone (P4) receptor antagonist, restrains the development and maturity of pinopodes (18). In the current study, the appearance of pinopodes following OS was earlier when compared with the control group, and the pinopode expression was restrained by mifepristone, which is consistent with the results of the aforementioned studies. Furthermore, previous studies on mice and humans have revealed that changes in the implantation window and abnormal expression of pinopodes may severely influence the synchrony between embryo and endometrial development, which is necessary for successful implantation (19,20). Therefore, the lower expression of fully developed pinopodes in the OS and EID model groups may be associated with the lower rates of pregnancy and embryonic implantation. The present study demonstrated that the spatial and temporal expression of pinopodes changes following OS or the administration of mifepristone, negatively impacting the synchrony between the embryo and endometrial development.

ZYR is a traditional Chinese medicine composed of *Epimedium brevicornum* Maxim, *Morinda officinalis* How, Chinese Dodder and *Eucommia ulmoides*. Although ZYR has been long used in infertility treatment, the underlying therapeutic mechanism has only been studied recently. ZYR has been found to attenuate the damage caused by superovulation and mifepristone by increasing the expression of endometrial LIF and integrin β3 subunit (9). In the present study, pinopodes were detected as a novel target of ZYR. The appearance of pinopodes in OS mice was delayed following treatment with ZYR, while the restrained expression of pinopodes in EID mice was improved following ZYR treatment, which were similar to the control mice. Previous studies have demonstrated that the duration of pinopode formation in rodent and human endometrium is limited to a short time period; in particular, pinopodes were found to persist in humans for <48 h during the mid-luteal phase of the menstrual cycle (15,16). In addition, pinopode appearance has been shown to be P4-dependent (21), while administration of estradiol (E_2_) results in the rapid loss of pinopodes (22). The P4 and E_2_ hormones act together to regulate the development and regression of pinopodes. Furthermore, oocyte donation studies have revealed that females treated with E_2_ and P4 have a higher chance of conception compared with individuals undergoing OS (15). In addition, the implantation window may be regulated by hormone replacement therapy in humans (23). Notably, pharmacological studies hypothesized that icarrin, *Morinda officinalis* How and Chinese Dodder have estrogen-like effects (24–26), while *Eucommia ulmoides* has progestin-like effects (27). Therefore, these observations led to hypothesis that the effect of ZYR on pinopode expression may be due to balancing the serum E_2_ and P4 levels in OS and EID mice, which regulate the spatial and temporal expression of pinopodes and synchronize the embryo and endometrial development at the time of implantation.

Controlled ovarian hyperstimulation treatment has been widely used as an important step in ART, aiming to increase the number of oocytes retrieved for IVF and improve the overall chance for a successful fertilization and pregnancy. However, due to the negative influence of superstimulation on endometrial receptivity, the pregnancy rate remains low, with the implantation of a high number of transferred embryos unsuccessful. Despite the considerable efforts of clinicians to improve endometrial receptivity by cryoperservation of embryos or local injury to the endometrium, the results remain unsatisfactory (28). In the present study, the significantly higher expression of pinopodes, increased pregnancy rate and increased number of embryonic implantation sites following treatment with ZYR reveals an alteration in the endometrial receptivity. Therefore, the results support the aforementioned hypothesis, and may provide further insight into the improved treatment of endometrial receptivity. As the effects of ZYR on the safety of offspring, and other molecular and genetic markers of endometrial receptivity in mice are currently unknown, further studies are required to provide more definitive answers.

In conclusion, the present study has revealed the beneficial effects of ZYR on pinopode expression on the endometrial surface of mice with EID and OS. The results demonstrated that treatment of EID and OS mice with ZYR significantly synchronized the expression of pinopodes and embryo development, and increased the number of embryo implantation sites and pregnancy rate. Therefore, ZYR may be used to improve endometrial receptivity and embryonic implantation in humans. Furthermore, the current study provides evidence on the mechanisms of traditional Chinese medicine, which may be used to safely circumvent the negative impact of OS on endometrial receptivity during the procedure of IVF. Thus, ZYR may provide a new insight into improving the treatment of endometrial receptivity. However, further studies are required to provide more information on the use of ZYR and address the safety of offspring.
